# Risk of treatment-altering haematological toxicity and its dependence on bone marrow doses in peptide receptor radionuclide therapy

**DOI:** 10.1186/s13550-024-01077-7

**Published:** 2024-02-06

**Authors:** Märta Persson, Cecilia Hindorf, Oscar Ardenfors, Martin Larsson, Joachim N. Nilsson

**Affiliations:** 1 Medicinsk Fysik och Teknik, Mälarsjukhuset, Eskilstuna, Sweden; 2 Department of Medical Radiation Physics and Nuclear Medicine, Karolinska University Hospital, Stockholm, Sweden; 3 Department of Molecular Medicine and Surgery, Karolinska Institutet, 17176 Stockholm, Sweden; 4 Department of Oncology-Pathology, Karolinska Institutet, Stockholm, Sweden; 5 Department of Endocrinology, Metabolism and Diabetes, Karolinska University Hospital, Stockholm, Sweden

**Keywords:** Dosimetry, PRRT, Neuroendocrine tumours, Bone marrow, Toxicity

## Abstract

**Background:**

Peptide receptor radionuclide therapy is effective in treating neuroendocrine tumours, but treatment may be limited by kidney and bone marrow toxicity. In this work, the absorbed dose burden to the bone marrow was estimated using image-based dosimetry and its potential use for predicting treatment-altering toxicity was studied. Peripheral blood samples taken before and after 229 treatments with ^177^Lu-DOTATATE in 59 patients were studied. In connection to the treatments, a total of 940 blood sample occasions provided data on white blood cell, neutrophil granulocyte, platelet, erythrocyte and haemoglobin concentrations. SPECT/CT image data were collected at two or three time points after each treatment. Absorbed doses to bone marrow were calculated from the activity concentration in a metastasis-free lumbar vertebra. The rate of delayed and aborted treatments was analysed based on medical records.

**Results:**

The average absorbed dose to the bone marrow was 0.42 Gy (median 0.33 Gy, SD 0.27 Gy) per treatment. Dose–response relationships between white blood cells, neutrophil granulocytes and haemoglobin concentrations were observed, most prominently at 31–45 days after each treatment. The correlations were stronger in patients with skeletal metastases. The rates of haematological toxicity-related delays and aborted treatments were 6% and 12%, respectively. None of the studied bone marrow dosimetric parameters could clearly predict treatment-related toxicity. However, patients with skeletal metastases had higher risk of treatment-altering toxicity (odds ratio = 6.0).

**Conclusions:**

Treatment-altering haematological toxicity in peptide receptor radionuclide therapy is relatively rare and appears difficult to fully predict from post-therapeutic image-based dosimetry. However, for patients with skeletal metastases, the haematological dose–response relationships are stronger. Future studies may focus on this patient group, to further investigate the usefulness of dosimetry in predicting decreases in blood values.

**Supplementary Information:**

The online version contains supplementary material available at 10.1186/s13550-024-01077-7.

## Background

Neuroendocrine tumours derive from cells in the neuroendocrine system that often express somatostatin receptors. In the last decade, the benefits of radionuclide therapy with somatostatin receptor-targeted ^177^Lu-DOTATATE peptide receptor radionuclide therapy (PRRT), have been established in clinical trials with improved survival and quality of life for patients with progressive or inoperable disease [1, 2]. While side effects of radionuclide therapy with ^177^Lu-DOTATATE are usually mild and manageable, one potential side effect is bone marrow suppression [3, 4], conferring higher risks of infection, treatment delay or abortion. Grade 3 or 4 haematological toxicity (Hb < 80 g/l, neutrophil granulocytes < 1 × 10^9^ cells/l, platelets < 50 × 10^9^ cells/l; CTCAE v5.0) is reported in some 5–10% of patients receiving radionuclide therapy with ^177^Lu-DOTATATE, making the bone marrow, together with kidneys, the main dose-limiting organs [3, 5–8]. Out of the haematological toxicities, suppressed neutrophil granulocytes may be the most clinically impactful, conferring higher risk for infection. In addition, 1–2% of patients receiving peptide receptor radionuclide therapy develop long-term haematological toxicity such as acute myeloid leukaemia and myelodysplastic syndrome, typically occurring 2–5 years after the end of treatment [5, 9]. In patients suffering from long-term haematological side effects of ^177^Lu-DOTATATE, an increase in mean corpuscular volume (MCV) of erythrocytes has been noted [3]. This increase could indicate an early sign of increased blood cell production and could therefore potentially be used as a marker in predicting short-term haematological toxicity.

Reported estimates of absorbed doses to the bone marrow from ^177^Lu-DOTATATE typically range between 0.1 and 0.4 Gy per infusion of 7.4 GBq [9–11]. Relationships between haematological toxicity and absorbed dose to the bone marrow, using a variety of different dosimetric methods, have been reported in previous publications [9, 12–14]. The reported data primarily concern dose–response relationships for platelet and white blood cell (WBC) concentrations, correlated both with mean and total absorbed dose. In another study, no correlation was found between the absorbed dose to the bone marrow and platelet counts [15].

This study specifically aimed to investigate if treatment-altering haematological toxicity could be predicted from the estimated absorbed dose to the bone marrow. A cohort with 59 patients having undergone frequent blood sampling is presented. An analysis based on MCV and neutrophil granulocytes was included, since to our knowledge, no such data have been previously published. Finally, the study aimed to find and further detail any relationships between absorbed dose to bone marrow and haematological toxicity.

## Material and methods

### Study conduct and inclusion criteria

Consecutive patients undergoing treatment with ^177^Lu-DOTATATE at Karolinska University Hospital, Stockholm, Sweden, between October 2019 and October 2022 were queried for study participation. All adult patients treated for any type of neuroendocrine tumour, neuroendocrine cancer or malignant pheochromocytoma were considered for study participation. In total, 69 patients were eligible. Written informed consent to participate and publish was obtained from participants, and the study was approved by the Swedish Ethical Review Authority (no. 2020-01541). Patients were excluded if only one post-treatment SPECT scan was available (4 patients), if only one treatment had been delivered (3 patients), if there was a severe lack of available blood sample data (2 patients, less than 2 samples per given treatment) or if absorbed dose to healthy bone marrow was impossible to evaluate (1 patient, due to pan-skeletal metastatic disease). In the resulting dataset, data from 940 blood sample time points in connection to 229 treatments in 59 patients were included. Patient characteristics are presented in Table 1. Blood sample data were collected up to December 2022. None of the included patients received blood transfusions during the study period. None of the patients had received ^90^Y-based therapy. However, nine of the included patients had received previous ^177^Lu-DOTATATE treatment at other hospitals (median time since last treatment 36 months). The previously treated patients were included, but detailed dosimetric and blood data from previous treatments were not available for analysis.

**Table 1:** Patient characteristics and treatment details

Parameter	Total (*n* = 59)
Age	
Median (range)	68 years (35–88)
Sex	
Male	35 (59%)
Female	24 (41%)
Subtype	
Gastrointestinal NET	45 (76%)
Pancreatic NET	8 (14%)
Lung NET	2 (3%)
Unspecified NET	2 (3%)
Pancreatic NEC	1 (2%)
Malignant pheochromocytoma	1 (2%)
‍Number of PRRT administrations per patient	
2	9 (15%)
3	6 (10%)
4	32 (52%)
5	9 (15%)
6	1 (2%)
7	1 (2%)
8	1 (2%)
Skeletal metastases	
Yes (proportion)	20/59 (34%)
1 lesion	1
2 lesions	4
3 lesions	2
4 lesions	0
≥ 5 lesions	13
Toxicity-related aborted treatment	
Yes (proportion)	7/59 (12%)
Previous PRRT	
Yes (proportion)	9/59 (15%)
Delay since previous treatment—median (range)	36 months (5–96)
Tumour grade	
Grade 1	18 (31%)
Grade 2	35 (59%)
Grade 3	4 (7%)
Unknown	2 (3%)

*NET* neuroendocrine tumour, *NEC* neuroendocrine cancer, *PRRT* peptide receptor radionuclide therapy

### Treatment with ^177^Lu-DOTATATE

^177^Lu-DOTATATE was administered as an intravenous infusion of 7.4 GBq per treatment, planned with intended intervals of 6–10 weeks, based on multidisciplinary tumour board decisions. Treatment interval intention was mainly based on tumour aggressiveness in terms of primary tumour origin, tumour grade and proliferation of the tumour cells (Ki-67 index). Treatments were given with concomitant amino acid infusion.

### Dosimetric analysis

**Fig. 1:**
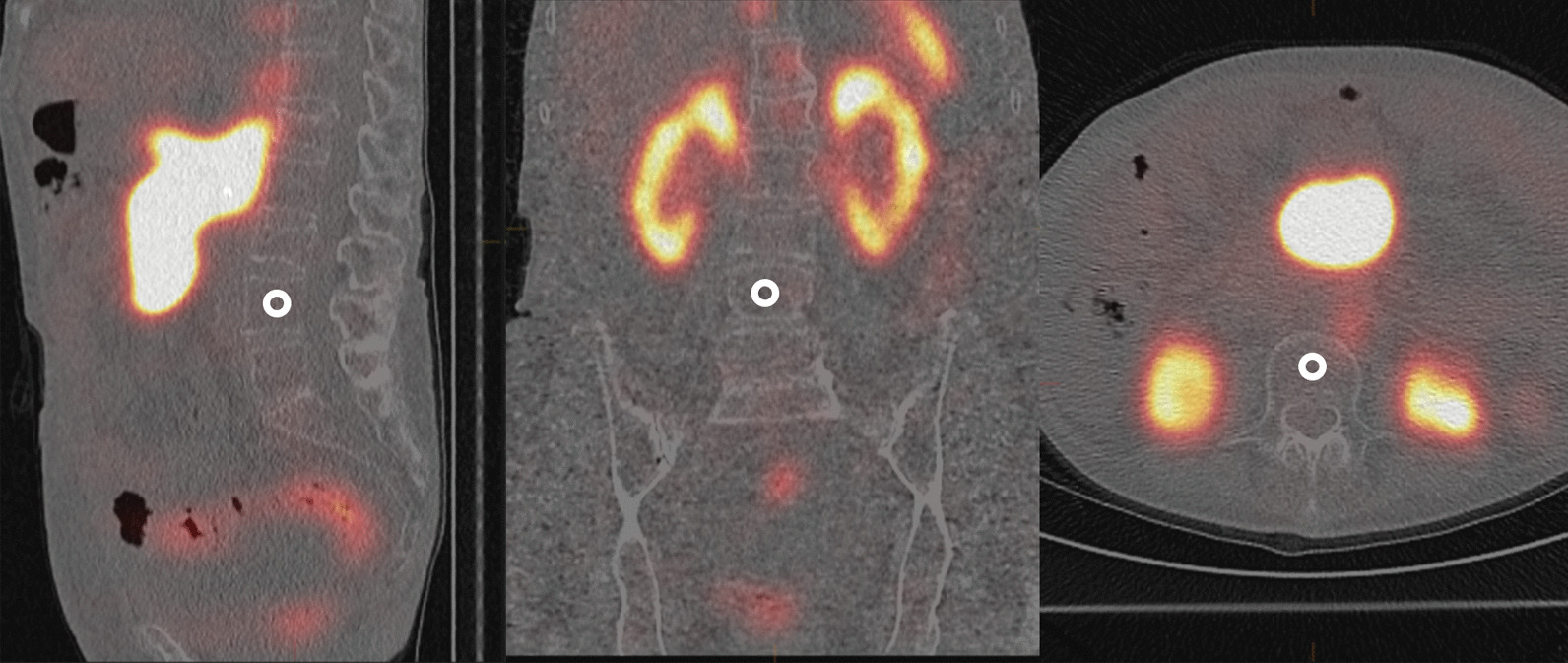
Location of the small spherical volume of interest in the L4 vertebra used to measure activity concentration. If skeletal metastases were present in L4, the volume of interest was placed in an adjacent vertebra (L3 or L5)

Absorbed doses were calculated by estimation of activity concentration in a small spherical volume of interest (approximately 0.5 cm^3^) on SPECT/CT data acquired at two or three time points post-administration (one, four and seven days post-administration). The volume of interest was placed within the vertebra in an area of homogeneous (low) activity, as shown in Fig. 1, which was considered representative of the concentration in the full vertebra. Only self-dose contribution was accounted for and any partial volume effect was considered negligible due to the homogeneous distribution. The measured activity concentration was used to determine absorbed dose to bone marrow by application of an activity concentration dose factor (ACDF), generated by in-house simulation. The method was based on a kidney dosimetry method previously described by Sandström et al. [11].

SPECT data were acquired with a total acquisition time of 20 min on GE 670 Discovery SPECT/CT systems, equipped with medium energy general purpose collimators. Images were reconstructed with ordered subset expectation maximisation (6 iterations, 10 subsets) using point spread function correction together with triple-window scatter correction and CT-based attenuation correction. The CT was also used for delineation. In order to validate the activity concentration-based methodology, absorbed doses to a subset of patients were studied with three other dosimetric methods, all showing similar results (see Additional file 1: S1). The differences between bone marrow absorbed dose estimates in different vertebrae in a subset of treatments were also performed (see Additional file 1: S1). The L4 vertebra was chosen as representative of the bone marrow based on a previous study on bone marrow toxicity [9]. Great care was taken to avoid sampling of any visible skeletal metastases, and baseline ^68^ Ga-DOTATOC PET/CT images were used to help determine this. If skeletal metastases were present in L4, the volume of interest was placed in L3 or L5. Mean and accumulated absorbed doses to the bone marrow were estimated for each administration of ^177^Lu-DOTATATE using an in-house dosimetry software to integrate a mono-exponential time-activity curve fitted to the activity concentrations in the spherical volume of interest. The activity concentration was approximated as constant between the time of administration and the first time point, after which a mono-exponential fit calculated from the first and all subsequent SPECT acquisitions was applied.

### Blood samples

Blood samples analysed for haemoglobin (Hb), WBC, erythrocytes, platelets, neutrophil granulocytes and MCV were routinely collected prior to the first ^177^Lu-DOTATATE administration as baseline. Blood samples were then collected at 1, 3, 5 and 7 weeks after each administration. Additional values were collected at the discretion of the treating physician beyond this time period if treatments were delayed or if it was the final administration, and blood samples up to six months after last treatment were included in the analysis. Further details on timing and distribution of blood samples can be found in Additional file 2: S2. Care was taken to exclude values from any significant transient medical causes such as treated infections and episodes of ileus. To account for individual variation in number of WBC and platelets, these values were normalised against individual baseline values (both prior to first treatment and prior to previous treatment, depending on analysis). If single blood values were missing, values were interpolated using nearest neighbour interpolation. For the first months of the study period (2019 and part of 2020), values of neutrophil granulocytes were only collected on clinical indication determined by the individual physician. Because of this, this period was excluded from the analysis of neutrophil granulocytes.

### Treatment-altering haematological toxicity

Any substantial delays in treatment administration (deviations of more than one week) from the intended treatment schedule, which was determined by a multidisciplinary tumour board prior to each treatment, were assessed. If any delays were found to be related to any cytopenia or anaemia, they were flagged as treatment-related delays in the analysis. The same analysis was performed for aborted treatments. The cohort was analysed retrospectively and the clinical decisions to abort or delay treatments, while not clearly predefined, were based mainly on levels of neutrophil granulocytes, in order to avoid risk of severe infections. These incidents constitute the basis for analysis of treatment-altering toxicity.

### Statistical analysis

All statistical analyses were performed using R (Version 4.2.2, R-project.org). Differences between dichotomous groups (e.g. absorbed doses in patients with delayed vs. non-delayed treatments) were tested for statistical significance using Welch's *t* test. Fisher's exact test was used when comparing number of delayed and aborted treatments between dichotomous groups. Linear regression and calculation of Pearson's correlation coefficient was performed between absorbed doses and all included blood parameters. Only moderate-to-strong correlation coefficients (greater than +/− 0.3) were considered potentially impactful and reported in the results section. When analysing the data for significant correlations, a *p* value of 0.01 was used, to avoid spurious results due to multiple testing. Prediction intervals were calculated (*lm* and *predict.lm* in R) to display the accuracy on any individual predictions based on the study data. To analyse if the absorbed dose to the bone marrow changed over the course of successive treatments, a mixed effects model (*lmer* in R) was used to separate patient variation from dependence on number of treatments. Power calculations were performed with the endpoint of predicting treatment-altering toxicity. The calculations assumed a baseline rate of 4% delayed or aborted treatments per administration, which is in the lower range of haematological toxicity rates reported in other studies [3, 5–8]. To detect an elevated rate of 15% (which was considered clinically significant) in a high-dose group with a statistical power of 80% would require 221 treatments (229 were eventually included in the dataset).

## Results

### Haematological effect of ^177^Lu-DOTATATE treatment

**Fig. 2:**
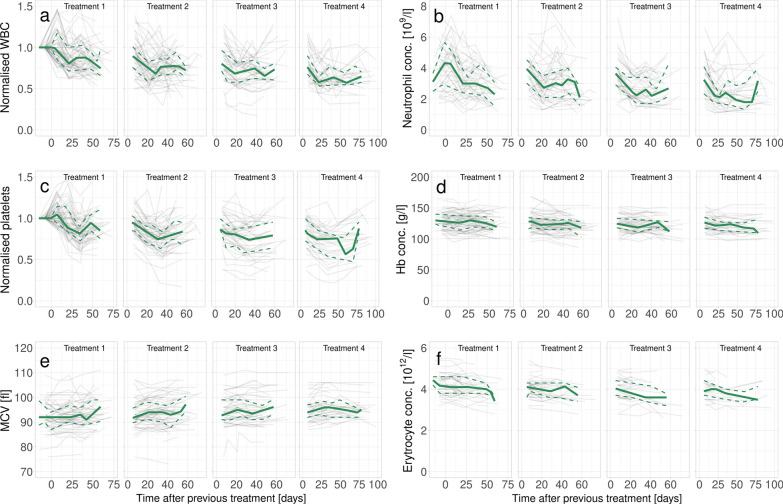
Changes in blood values over the course of the first four treatments with 177-Lu-DOTATATE. The solid green curve represents the median and the dashed lines represent the 25 and 75 percentiles, respectively. Grey lines show the values of each patient. **a** Normalised WBC concentration, **b** absolute neutrophil concentration, **c** normalised platelet concentration, **d** absolute Hb concentration, **e** MCV on a nonzero scale and **f** absolute erythrocyte concentration

Overall changes in blood values, normalised to baseline values before first treatment, as function of time from the previous treatment are shown in Fig. 2, for each treatment with ^177^Lu-DOTATATE. Patient characteristics are presented in Table 1.

**Fig. 3:**
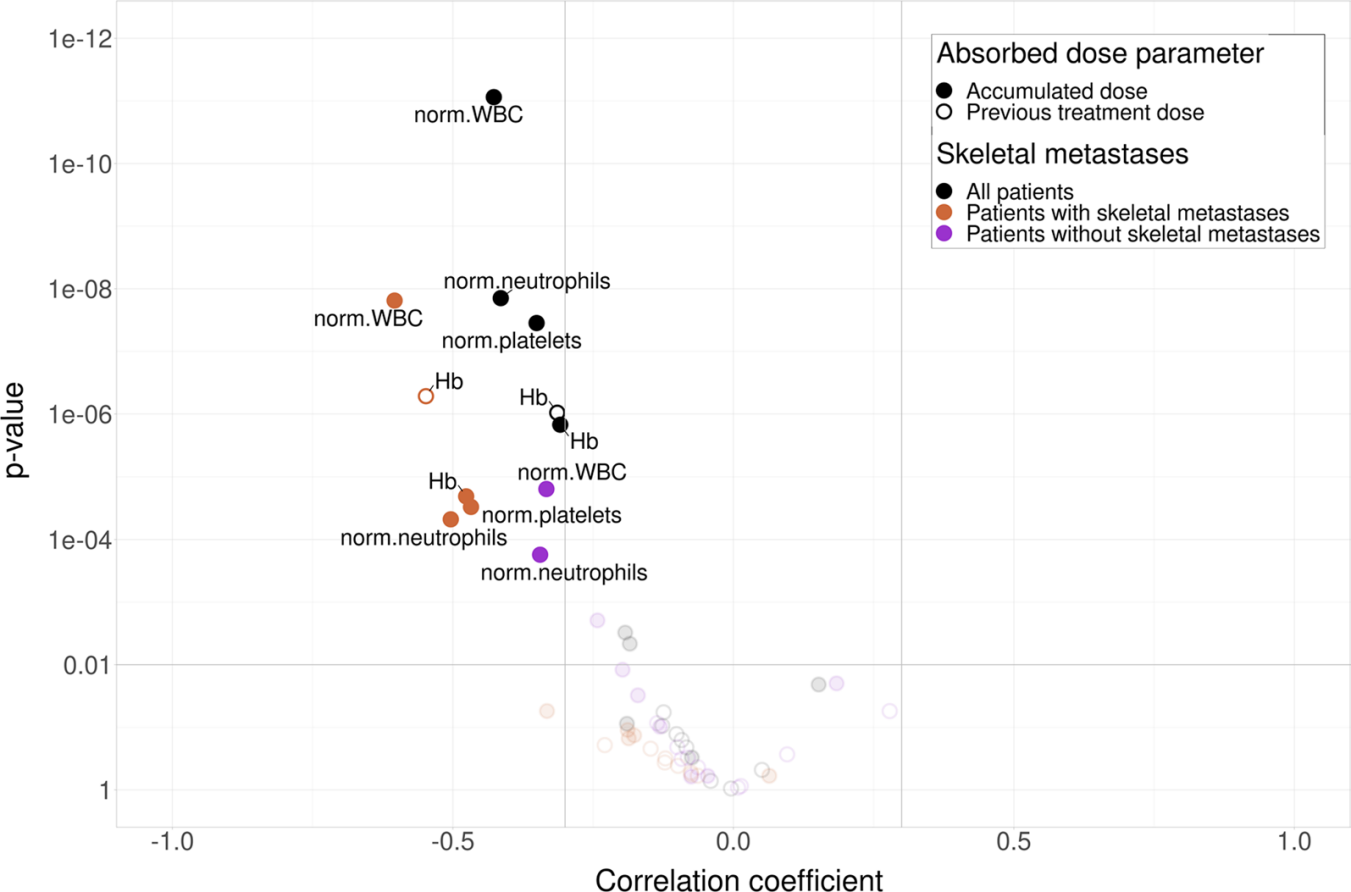
Dose–response relationships between blood samples (white blood cells—WBC, platelets, neutrophil granulocytes and Hb) and absorbed doses to bone marrow, presented as a volcano plot. Data are shown for blood samples collected 31–45 days after each treatment. While correlations were found for all patients (black markers), the correlations were generally stronger in patients with skeletal metastases (orange markers) compared to those without (purple markers). Only Hb showed a correlation between change in concentration and absorbed dose from previous treatment (open circle markers). All other correlations were for cumulated absorbed dose. Markers with low confidence (*p* values > 0.01) and weaker correlations than 0.3 are greyed out

Correlations between absorbed dose and blood counts were compared for different blood sampling intervals (at 1–15, 16–30, 31–45, 46–60, 61–75, 76-days after treatment). Significant correlations (*p* < 0.01) were found for WBC, neutrophil granulocytes, Hb and platelets. While most of the correlations were observed in several of the blood sampling intervals, the time interval in which correlations were strongest was between 31 and 45 days after treatment administration, as can be seen in Fig. 3. Correlations between WBC, neutrophil granulocytes, Hb and platelets were found for all patients, but the correlations were generally stronger in patients with skeletal metastases compared to those without. Only Hb showed a correlation between change in concentration and absorbed dose from previous treatment. All other correlations were for cumulated absorbed dose. The median nadirs were found within this time interval (35 days after treatment) for WBC, neutrophil granulocyte and platelet count (IQR (21–49), (21–54) and (28–36), respectively). Data for all blood sampling intervals, and correlation for the final treatment for each patient, are presented in Additional file 3: S3.

### Dose–response relationships

**Fig. 4:**
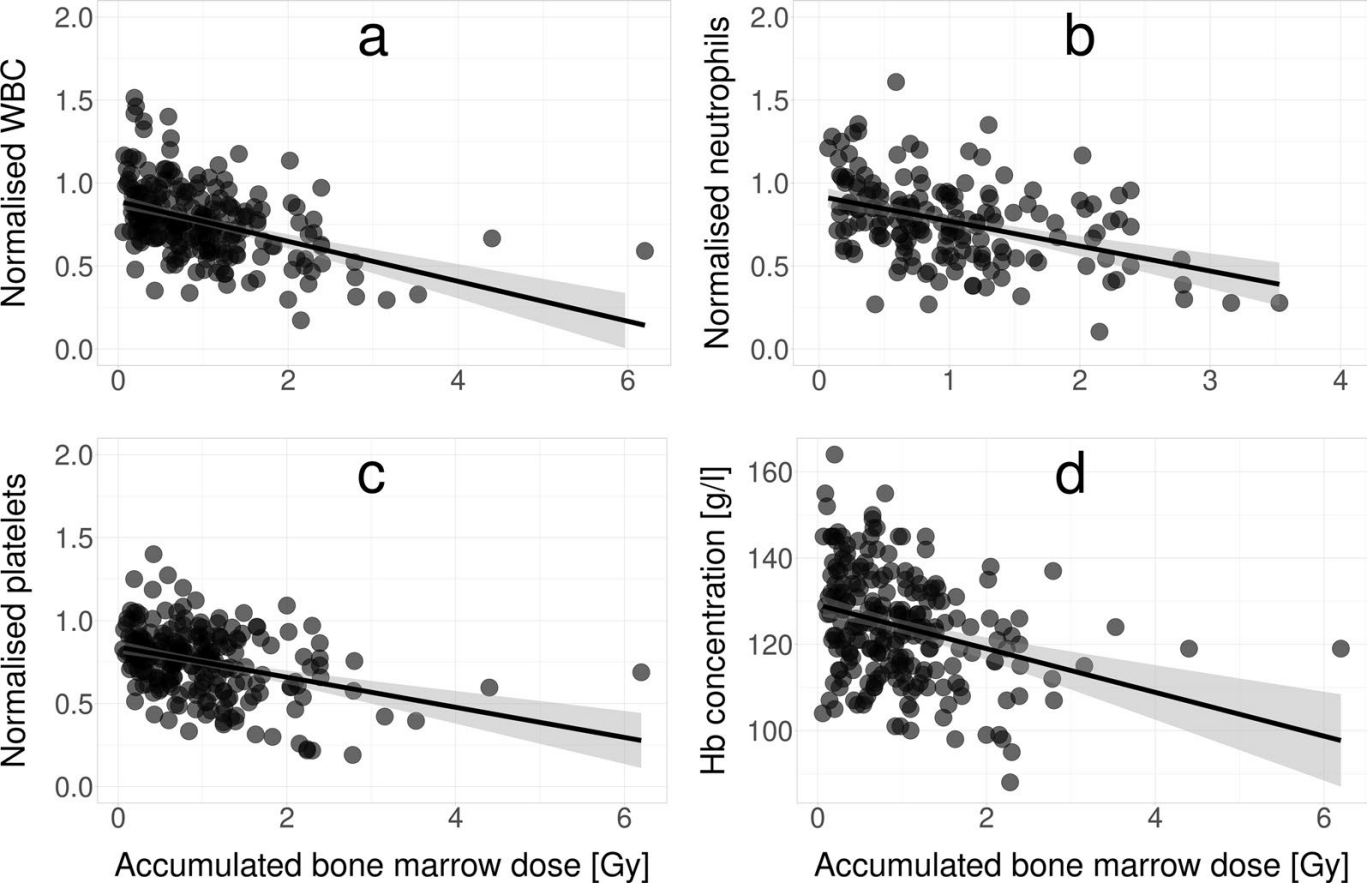
Dose–response relationships between **a** normalised white blood cell (WBC), **b** normalised neutrophil granulocyte, **c** normalised platelet and **d** absolute haemoglobin (Hb) concentrations and accumulated absorbed dose (from all given treatments combined). All shown blood data were sampled 31–45 days after administration of 177-Lu-DOTATATE. Markers are transparent so that overlapping points are resolved as darker markers. The linear fits are shown as lines along with the corresponding confidence intervals of the fits (shaded area)

Significant correlations were found between accumulated absorbed dose (absorbed dose from all given treatments combined) and normalised WBC (*r* = −0.43, CI − 0.32 to − 0.53), normalised neutrophil granulocytes (*r* = −0.42, CI − 0.28 to − 0.53), normalised platelets (*r* = −0.35, CI − 0.23 to − 0.46) and Hb (*r* = −0.31, CI − 0.19 to − 0.42), as shown in Fig. 4.

For WBC, neutrophil granulocytes and platelets, an approximate 35% decrease was observed at an accumulated absorbed dose of 2 Gy (WBC: − 35%, prediction interval − 74% + 4%; neutrophil granulocytes: − 38%, prediction interval − 83% to + 7%; platelets: − 34%, prediction interval − 71% to + 3%). For Hb, a change of − 5 g/l per 2 Gy (prediction interval − 29 g/l to + 19 g/l) was observed. The large variation between patients, and corresponding wide prediction intervals, can be seen in Fig. 4. No significant correlations were found between MCV or erythrocytes and absorbed doses to bone marrow. There are correlations between absorbed doses from the immediately previous treatment and blood level changes after that treatment was only found for Hb (*r* = − .31, CI − 0.19 to − 0.43). Separating data according to presence of skeletal metastases revealed that most observed correlations were stronger in patients with skeletal metastases. In patients without skeletal metastases, statistically significant correlations with absorbed dose were only found for normalised WBC and neutrophil granulocytes. Absolute neutrophil granulocyte count was not significantly correlated with absorbed dose to the bone marrow (*r* = −0.07, CI − 0.21 to 0.07).

### Absorbed doses

**Fig. 5:**
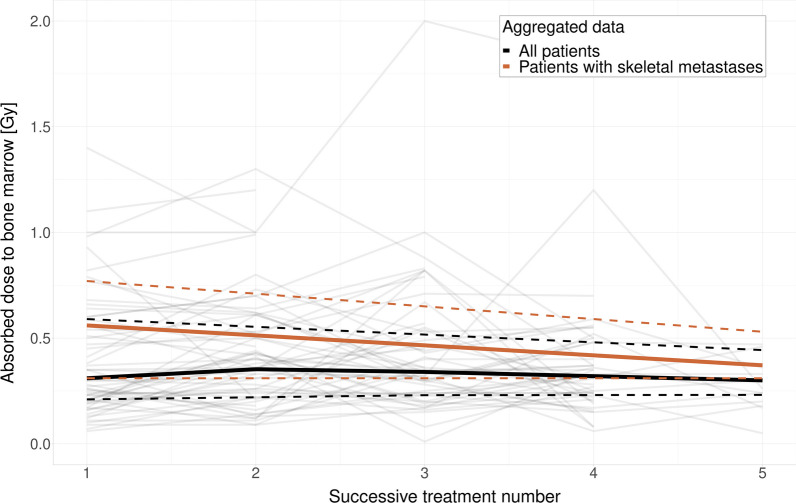
Absorbed dose to the bone marrow over the course of all received treatments for all patients (black curve) and patients with skeletal metastases (orange curve). No significant change in absorbed dose for each treatment was observed in either patient subgroup. The solid curves represent the median and the dashed lines represent the 25 and 75 percentiles, respectively. Grey lines show the individual values for each patient. Only three patients received more than five treatments—those data points are not shown in this graph

No increase in absorbed doses to the bone marrow per treatment was observed over the course of the administered treatments, as shown in Fig. 5. The average absorbed dose to the bone marrow from all treatment cycles for all patients was 0.42 Gy (standard deviation: 0.27 Gy). Patients with skeletal metastases received 0.20 Gy (CI 0.06 to 0.35 Gy) higher absorbed dose to the bone marrow on average (0.56 vs 0.36 Gy for patients with and without skeletal metastases). No significant change in intra-patient absorbed dose was observed over successive treatments (0.00 change in Gy/treatment, CI − 0.02 to 0.02). The effective half-life of activity in the studied non-metastatic vertebrae for the population was estimated to a median of 88 h (mean 90 h, standard deviation 25 h, range 46–155 h).

### Risk of delayed and aborted treatment

Treatment series were aborted due to treatment-related haematological toxicity in 12% (7/59) of patients. Administration of ^177^Lu-DOTATATE was delayed due to transient haematological toxicity in 6% (13/229) of treatments. Absorbed doses to the bone marrow (either from previous treatment or accumulated to the delayed treatment) were not significantly higher in patients that had delays than in patients receiving treatments as planned. No significant difference was found in baseline WBC, neutrophil granulocyte and platelet concentrations between patients that went on to have aborted treatments and those who received all treatments with the intended interval. No patients had baseline WBC or neutrophil granulocyte concentrations below the lower normal reference level at the start of treatment.

Patient age was not significantly higher for patients with aborted treatments (CI − 1.5 to 10.6 years). The only observed significant difference between patients who had aborted treatments and others was the presence of skeletal metastases (odds ratio 6.0, *p* value 0.038). A separate analysis was conducted by excluding data from the nine patients that had received previous ^177^Lu-DOTATATE treatments, with detailed results shown in Additional file 4: S4. Similar correlations were found for normalised WBC, neutrophil granulocyte and platelet concentrations when excluding previously PRRT-treated patients. Furthermore, patients that had received previous treatment were not at greater risk of delayed or aborted treatment.

## Discussion

The presented data strengthen some already published data on the link between absorbed doses and bone marrow suppression. Bergsma et al. reported correlations between bone marrow absorbed doses (determined from blood and urine samples in combination with planar image-based dosimetry) and decreased WBC and platelet concentrations in 32 patients [10]. Svensson et al., using image-based dosimetry with both planar and SPECT/CT data, similarly found a significant absorbed dose correlation for platelets and WBC concentrations in 46 patients, while also reporting a significant correlation between absorbed dose and Hb concentration [12], while based on a different dosimetric approach, our study confirms those relationships, while also adding data on neutrophil granulocytes. The current results also align well with the observed trends in platelets and Hb reported by Minczeles et al. [16]. The dataset of the presented study is larger than those previously published with dosimetric data and may therefore have higher statistical power to find any underlying dose–response relationships.

Neutropenia, arguably the most clinically impactful toxicity in ^177^Lu-DOTATATE, was not readily predicted by absorbed dose in the current dataset. While a dose–response relationship was found for normalised neutrophil concentrations, a significant correlation was not observed in absolute values, and no patients fell below the threshold for grade 3 neutropenia (< 1 × 10^9^ cells/l; CTCAE v5.0).

The overall concentrations of the measured blood cells tended to decline over time as the patients received higher accumulated activities. However, no early onset toxicity seemed likely, as was evident by the low rate of toxicity-related delays (6% of administrations) in the current dataset. This finding supports the suggestion by Bergsma et al. that an accumulated absorbed dose limit of 2 or 3 Gy to the bone marrow in PRRT is probably overly conservative [10]. This is to some degree supported by use of ^131^I in thyroid cancer treatment, where 2 Gy to blood per treatment (used as a proxy for bone marrow absorbed dose) can be reached for elderly patients, with little substantial haematological toxicity even over repeated treatments [17, 18]. While there is a known saturation effect at 2 Gy for blood cells, as determined from external beam radiation with higher dose rates [19], the dose rate and fractionation in PRRT probably allows for a higher cumulative dose burden without high risk of toxicity. The infrequent occurrence of treatment-altering haematological toxicity limits the value of bone marrow dosimetry as a routine procedure for all patients receiving treatment according to the standard schedule (7400 MBq administered with intervals of 6–10 weeks); limited health-care resources may be more usefully allocated elsewhere. However, the results indicate that a narrowed approach can be fruitful: in patients with skeletal metastases, the odds ratio for aborted treatments was found to be sixfold. Since both the risk of aborted treatments and dose–effect correlations were largest in patients with skeletal metastases, dosimetry-based guidance for treatment length or spacing may have higher value in this subgroup of patients. However, it is not yet clear how such a scheme should best be devised. Skeletal metastases have been reported as a risk factor for bone marrow toxicity by others [10, 13, 16], but with some contradictory data [6], that reported no higher risk of subacute haematological toxicity in patients with skeletal metastases. Bodei et al. have previously reported risk factors for long-term haematological toxicity, including initial thrombocytopenia and prior chemotherapy, which was not given to any patients in the current dataset [7]. A limiting factor in this study is the absence of data on total tumour burden per treatment, due to only one field of SPECT/CT being available for analysis. Total tumour burden has previously been noted to predict toxicity and can alter kinetics of ^177^Lu-DOTATATE significantly [10].

The reason for why absorbed dose–relationships are stronger in patients with skeletal metastases is unclear from our presented data. The applied methodology avoided any measurement of vertebrae that had visible bone marrow metastases. However, considering the limited sensitivity and spatial resolution of the available SPECT/CT and PET/CT, this cannot be asserted with full certainty. A biological cause for the stronger dose–response relationship in patients with skeletal metastases may be that those patients have less healthy bone marrow reserve that can act as a buffer, causing the observed linear dose–response relationships to be more prominent. Another possible explanation is that patients with known skeletal metastases elsewhere are more likely to have small metastases in vertebrae that were analysed in this work. Such metastases may have both contributed to the higher absorbed doses found in those patients, but may also be associated with confounding factors that in themselves make bone marrow toxicity more likely. Any such confounders may explain the stronger correlations found between absorbed dose and toxicity for patients with skeletal metastases. The absorbed doses to bone marrow in this work (mean 0.42 Gy/7.4 GBq treatment) are however within the previously reported range of bone marrow doses, albeit in the upper quartile of reported values [9–11].

In this work, no predictive value of MCV was found. While a slow increase over the course of multiple treatments was observed (see Fig. 2), it did not appear to predict haematological toxicity earlier than decreases in blood counts.

The dosimetry method used in this work is a simplified way of estimating the full activity content in the bone marrow by sampling only one lumbar vertebrae. Any imprecision in the determination of true absorbed dose to the bone marrow is likely to obscure the relationships studied in this work. Several dosimetric methods were explored in the early parts of this work, both *S*-value-based, activity concentration-based and dose-kernel-based calculations, the results of which are detailed in Additional file 1: S1. However, no significant differences in absorbed doses were observed. This is similar to the study by Hagmarker et al. [9], which found dose–response relationships independently of which vertebra (or if all visible vertebrae) was studied. Other studies have validated hybrid image-based bone marrow dosimetry methods, further supporting the dosimetric approach used in the present study [20]. Also, recent data indicating a specific bone marrow uptake of ^177^Lu-DOTATATE emphasise the importance of image-based dosimetry methods [21].

No difference in risk of treatment-altering toxicity was observed in the subset of patients that had received previous cycles of PRRT, in line with previous data reported by van Essen et al. [22]. However, the retrospective nature of this study could indicate a selection bias, since only relatively fit patients with well-functioning bone marrow went on to receive treatment in the presented dataset, limiting this to a light observation.

## Conclusions

Treatment-altering short-term haematological toxicity in PRRT is relatively rare and appears difficult to fully predict from post-therapeutic bone marrow dosimetry. However, for patients with skeletal metastases, in whom haematological toxicity is more common, the haematological dose–response relationships are stronger. Future studies may focus on this patient group, to further investigate the usefulness of dosimetry in predicting future decreases in blood values.

### Supplementary Information

**Additional file 1.** Bone marrow dosimetry methods.

**Additional file 2.** Distribution of blood sample time-points.

**Additional file 3.** Volcano plot montage.

**Additional file 4.** Correlation analysis with and without previously treated patients.

## Data Availability

The datasets used and analysed during the current study are available from the corresponding author on reasonable request.

## Abbreviations

ACDF: Activity concentration dose factor
CT: Computed tomography
CTCAE: Common terminology criteria for adverse events
DOTATATE: DOTA-(Tyr^3^)-octreotate
Hb: Haemoglobin
MCV: Mean corpuscular volume
NET: Neuroendocrine tumour
NEC: Neuroendocrine cancer
PET: Positron emission tomography
PRRT: Peptide receptor radionuclide therapy
SPECT: Single-photon emission computed tomography
WBC: White blood cells
