# Supplementary material for: Risk of treatment-altering haematological toxicity and its dependence on bone marrow doses in peptide receptor radionuclide therapy
Source: EJNMMI Res. 2024 Feb 6;14:13. doi: 10.1186/s13550-024-01077-7 (PMC10847080; doi:10.1186/s13550-024-01077-7)
Supplement: Supplementary file 1 — Additional file 1. Bone marrow dosimetry methods. [file 13550_2024_1077_MOESM1_ESM.pdf]

Supplementary material – Bone marrow dosimetry

Background/Methodology

Activity concentration-based dosimetry was performed by measuring activity concentration in the whole L4 vertebra on SPECT/CT data aquired at two or three time points post administration (one, four and seven days post-administration). The measured activity concentration was used to construct a time-activity curve, which was integrated to obtain the cumulated activity, Ã. Assuming that any cross absorbed dose contribution was negligible and most of the emitted energy absorbed locally, the absorbed dose was obtained by multiplication with an appropriate S-value (see equation below), obtained from a curve fitted to S-values generated by in-house simulations for various volumes of a water sphere as a function of sphere volume. If the volumes of interest were not equal in size, their average was used to obtain S-values from the curve fit.

$$D[Gy]=\tilde{A}[MBq\cdot s]\times S_{BM\rightarrow BM}[Gy/MBq\cdot s]$$

A similar method was applied using an activity concentration measured in a small volume within the vertebra in an area of homogeneous activity uptake which was considered representative of the concentration in the full vertebra, a method developed based on the works of Sandström et. al. [1] as well as Hagmarker et. al. [2]. Instead of applying an S-value an Activity Concentration Dose Factor (ACDF) was used (see equation below), which was generated by in-house simulations of ^{177}Lu in water using the software IDAC [3].

$$D[Gy]=\tilde{A}_{conc}[MBq\cdot s/ml]\times ACDF[Gy\cdot ml/MBq\cdot s]$$

In addition, a kernel-based approach to bone marrow dosimetry was performed. In contrast to the activity concentration-based methods, this method included contribution from cross absorbed dose from adjacent high-uptake organs and malignancies (within approximately 10 cm from the volume of interest). A 45x45x45 pixel (approximately 20x20x20 cm) kernel was applied to the SPECT data, creating a dose rate map from which absorbed dose to the bone marrow was measured in the whole vertebra. The dose kernel was water-based and had been previously generated by in-house simulations using MCNP6.

Due to low uptake of the radionuclide it was not possible to locate the vertebrae on the SPECT images, so for all dosimetry methods the VOI was positioned with guidance from CT-data acquired in conjunction with the SPECT acquisition.

Partial volume effect was considered negligible in all three methods due to homogeneous uptake in the vertebrae and surrounding areas.

Results

The three dosimetry methods generated similar estimates of absorbed dose to the bone marrow (per treatment and in total) which were all well below the commonly accepted limit of 2 Gy [4]. Values are presented in Table 1 as Mean ± Coefficient of Variation (CV). Figure 1 displays the mean absorbed dose to the bone marrow per treatment for each patient included in the analysis. Bland-Altman plots for the small volume method vs. all others are shown in Figure 2. As seen in the figure, only for a few patients does the estimated absorbed dose to the bone marrow differ notably between the different dosimetry methods. In terms of correlations with declining blood cell counts all three methods also generated similar results.

Absorbed doses in L3 tended to be higher than in L4 and L5 when comparing in a subset of treatments, see Figure 3. While there was some difference between doses to the different vertebrae, it is not possible to determine if this is is due to biological reasons or measurement uncertainty. The overall trends over treatments for the three patients studied are similar for the different vertebrae, indicating that it is not a purely random variation.

Table 1: Absorbed dose to the bone marrow (per treatment and in total) determined with the three respective dosimetry methods, expressed as Mean ± Coefficient of Variation (CV).

	Absorbed dose to the bone marrow per treatment (Gy per 7.4 GBq)	Total absorbed dose to the bone marrow (Gy)
Activity concentration (Small volume) method	0.39 ± 0.87	1.29 ± 0.64
Activity concentration (Whole vertebra) method	0.35 ± 1.07	1.13 ± 0.80
Dose-kernel method	0.38 ± 0.87	1.25 ± 0.77

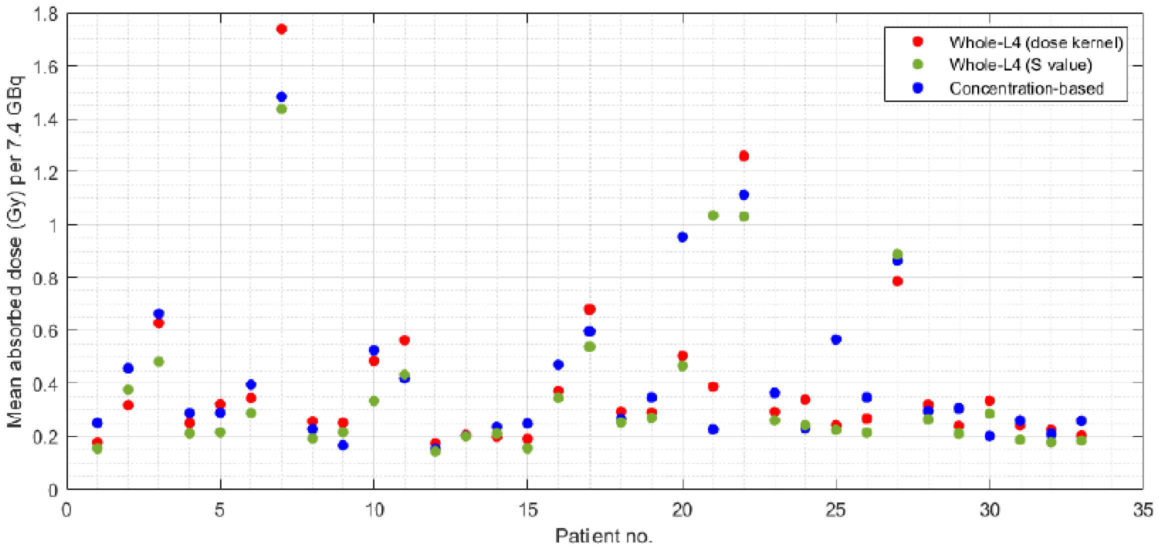

Figure 1: Mean absorbed dose to the bone marrow (Gy) per 7.4 GBq 177Lu-DOTATATE for each patient. The colors indicate the applied dosimetry method (red = dose-kernel, green = activity concentration (full vertebra), blue = activity concentration (small volume))

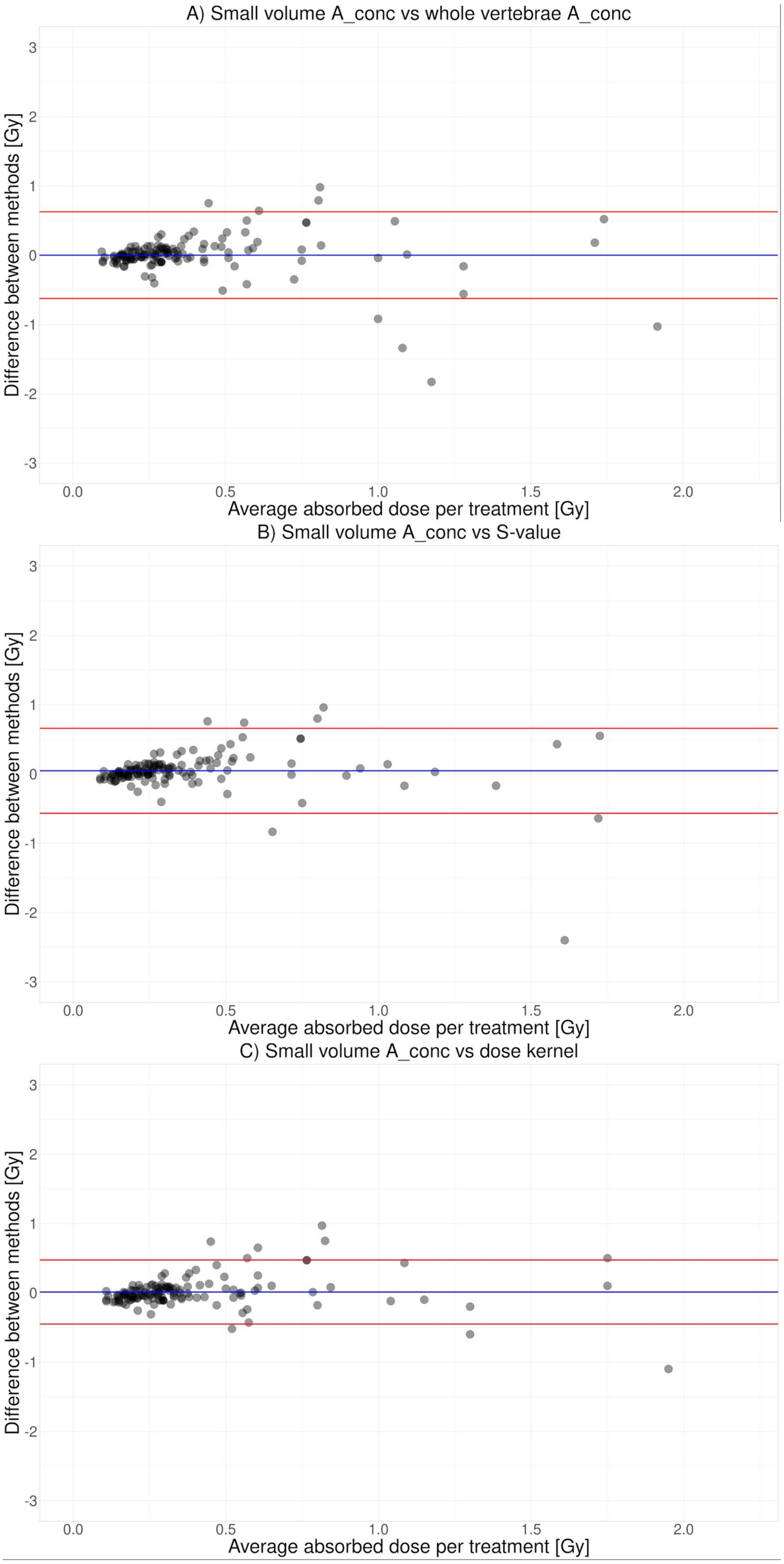

Figure 2: Bland-Altman plots for the small volume ROI estimating activity concentration vs. the other dosimetric methods; A) whole vertebrae activity concentration, B) S-value for water, C) dose-kernel convolved images with applied recovery coefficients.

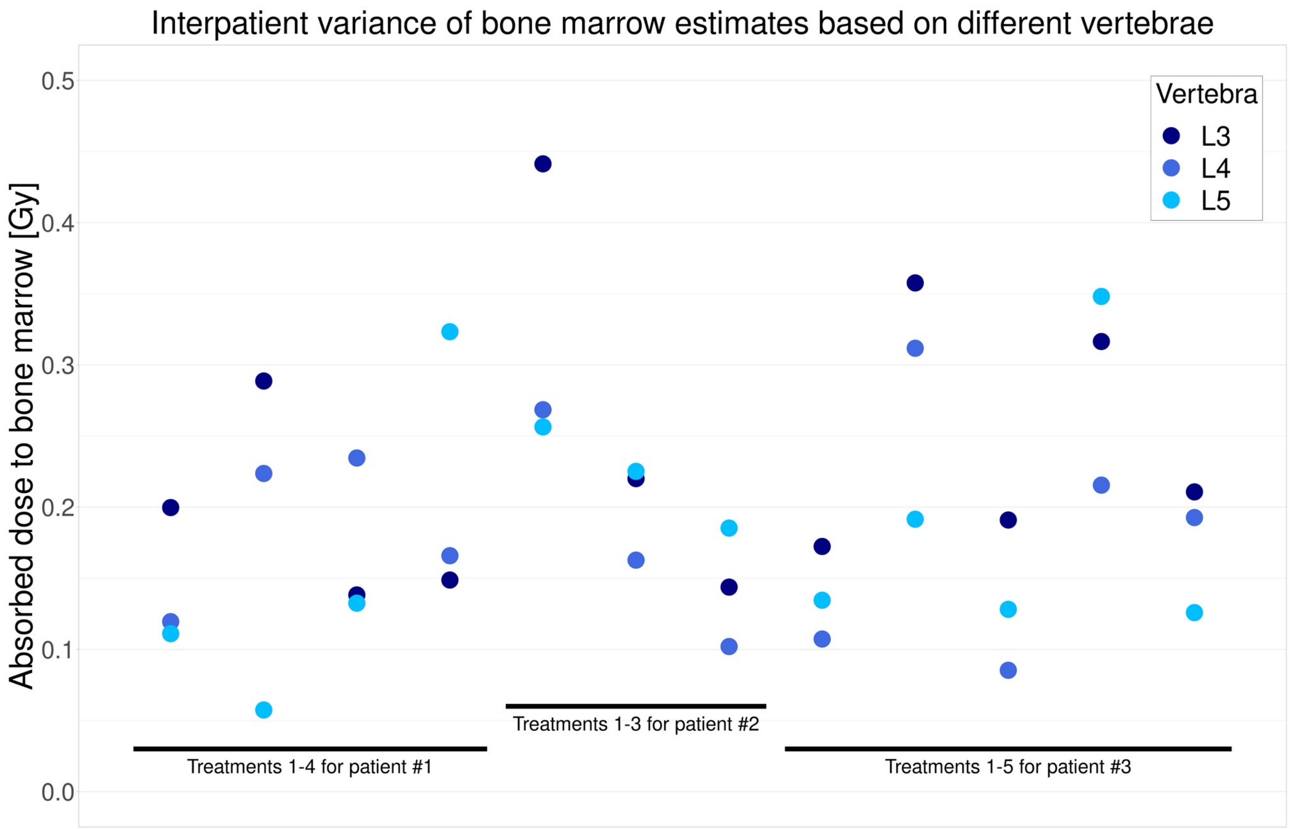

Figure 3: Comparison of bone marrow doses to three different vertebrae (L3, L4 and L5) from 12 treatments in three different patients.

References

1. Sandström, M.; Garske-Román, U.; Granberg, D.; Johansson, S.; Widström, C.; Eriksson, B.; Sundin, A.; Lundqvist, H.; Lubberink, M. Individualized Dosimetry of Kidney and Bone Marrow in Patients Undergoing 177Lu-DOTA-Octreotate Treatment. *Journal of Nuclear Medicine* **2013**, *54*, 33–41, doi:10.2967/jnumed.112.107524.
2. Hagmarker L; Svensson J; Rydén T; van Essen M; Sundlöv A; Gleisner Ks; Gjertsson P; Bernhardt P Bone Marrow Absorbed Doses and Correlations with Hematologic Response During 177Lu-DOTATATE Treatments Are Influenced by Image-Based Dosimetry Method and Presence of Skeletal Metastases. *Journal of nuclear medicine : official publication, Society of Nuclear Medicine* **2019**, *60*, doi:10.2967/jnumed.118.225235.
3. Andersson, M.; Johansson, L.; Eckerman, K.; Mattsson, S. IDAC-Dose 2.1, an Internal Dosimetry Program for Diagnostic Nuclear Medicine Based on the ICRP Adult Reference Voxel Phantoms. *EJNMMI Res* **2017**, *7*, 88, doi:10.1186/s13550-017-0339-3.
4. Svensson J; Rydén T; Hagmarker L; Hemmingsson J; Wängberg B; Bernhardt P A Novel Planar Image-Based Method for Bone Marrow Dosimetry in (177)Lu-DOTATATE Treatment Correlates with Haematological Toxicity. *EJNMMI physics* **2016**, *3*, doi:10.1186/s40658-016-0157-0.
