# Supplementary material for: Risk of treatment-altering haematological toxicity and its dependence on bone marrow doses in peptide receptor radionuclide therapy
Source: EJNMMI Res. 2024 Feb 6;14:13. doi: 10.1186/s13550-024-01077-7 (PMC10847080; doi:10.1186/s13550-024-01077-7)
Supplement: Supplementary file 2 — Additional file 2. Distribution of blood sample time-points. [file 13550_2024_1077_MOESM2_ESM.pdf]

Supplementary material - Distribution of blood sample time-points

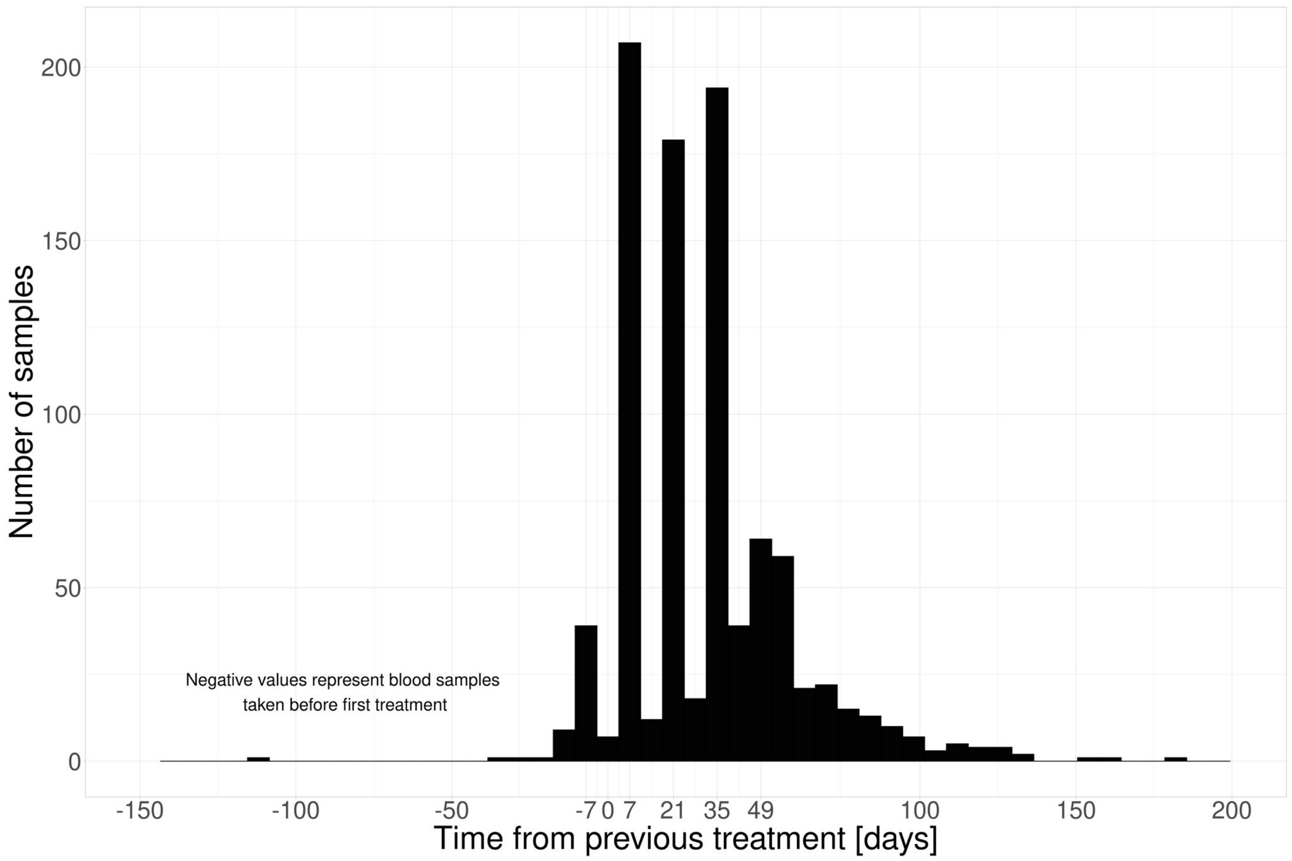

Figure 1: Time-points of blood samples. Routine samples were collected at -1, 1, 3, 5 and 7 weeks (-7, 7, 21, 35 and 49 days) post-treatment. Further blood samples were collected after last treatment and in case of delayed treatments. The outlier at -111 days was a patient that had blood samples taken at another institution, not available for analysis within the study. The previously available blood values at our institution taken 111 days before treatment was used instead.
