# Supplementary figures and images for: Risk of treatment-altering haematological toxicity and its dependence on bone marrow doses in peptide receptor radionuclide therapy
Source: EJNMMI Res. 2024 Feb 6;14:13. doi: 10.1186/s13550-024-01077-7 (PMC10847080; doi:10.1186/s13550-024-01077-7)

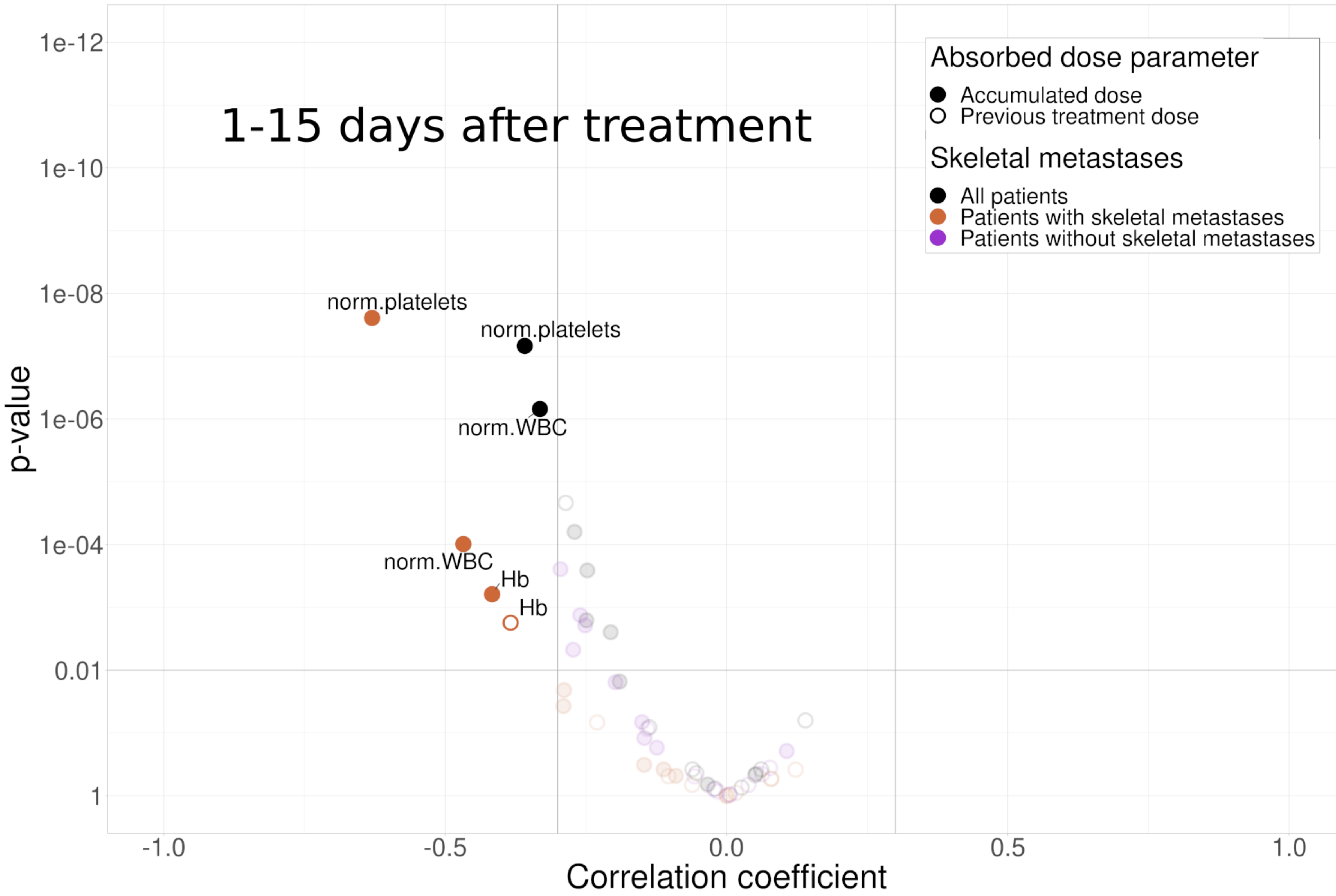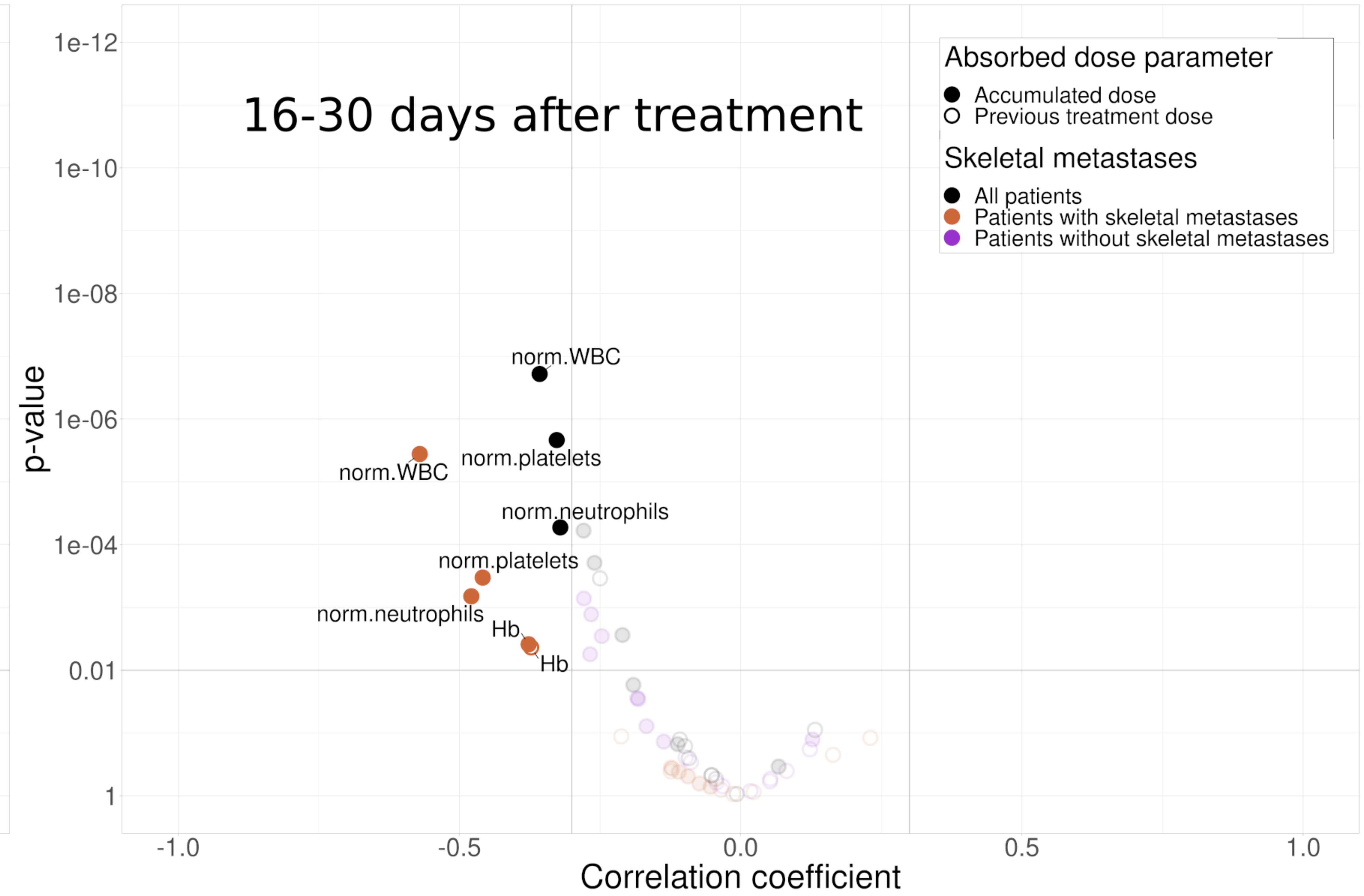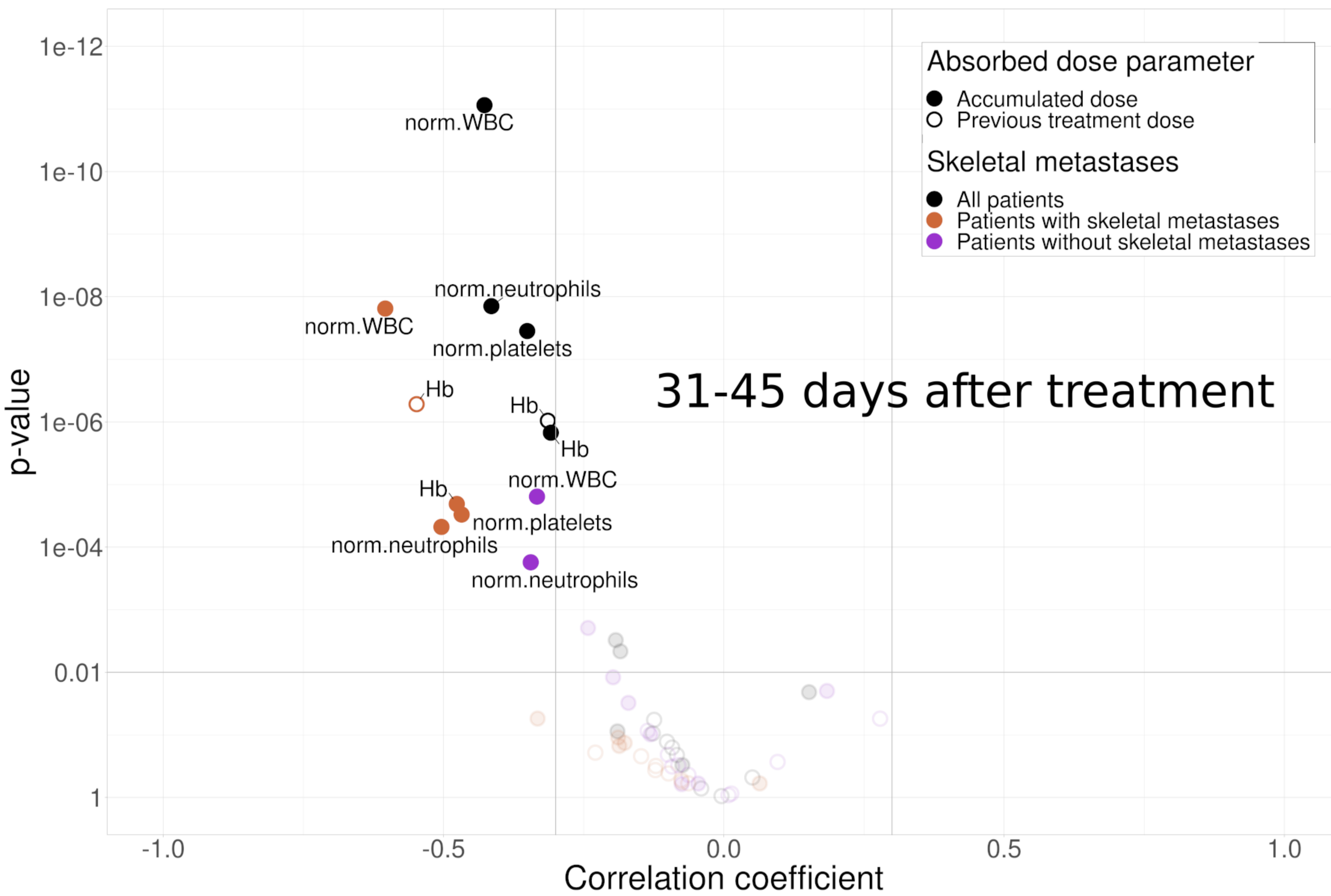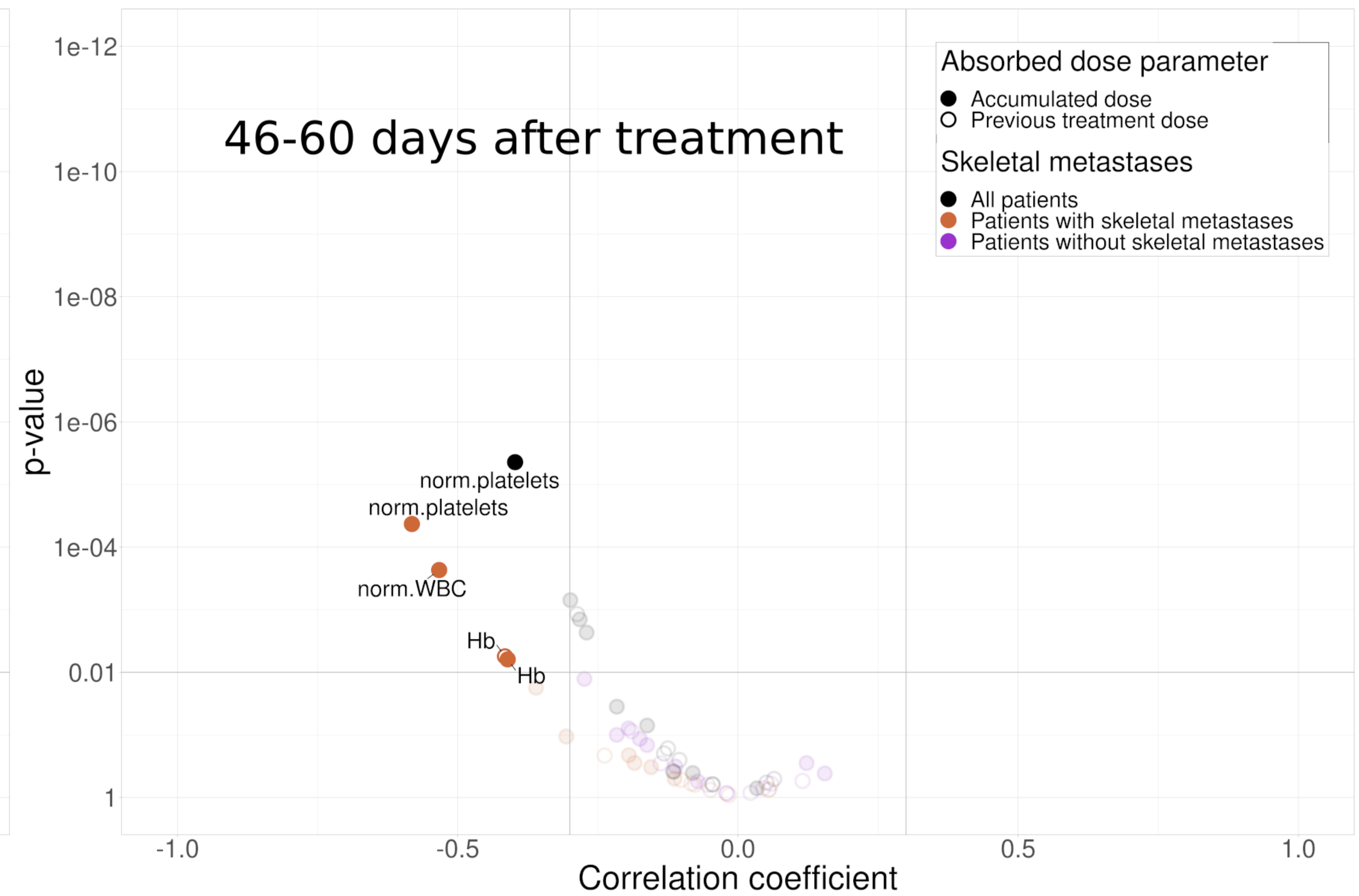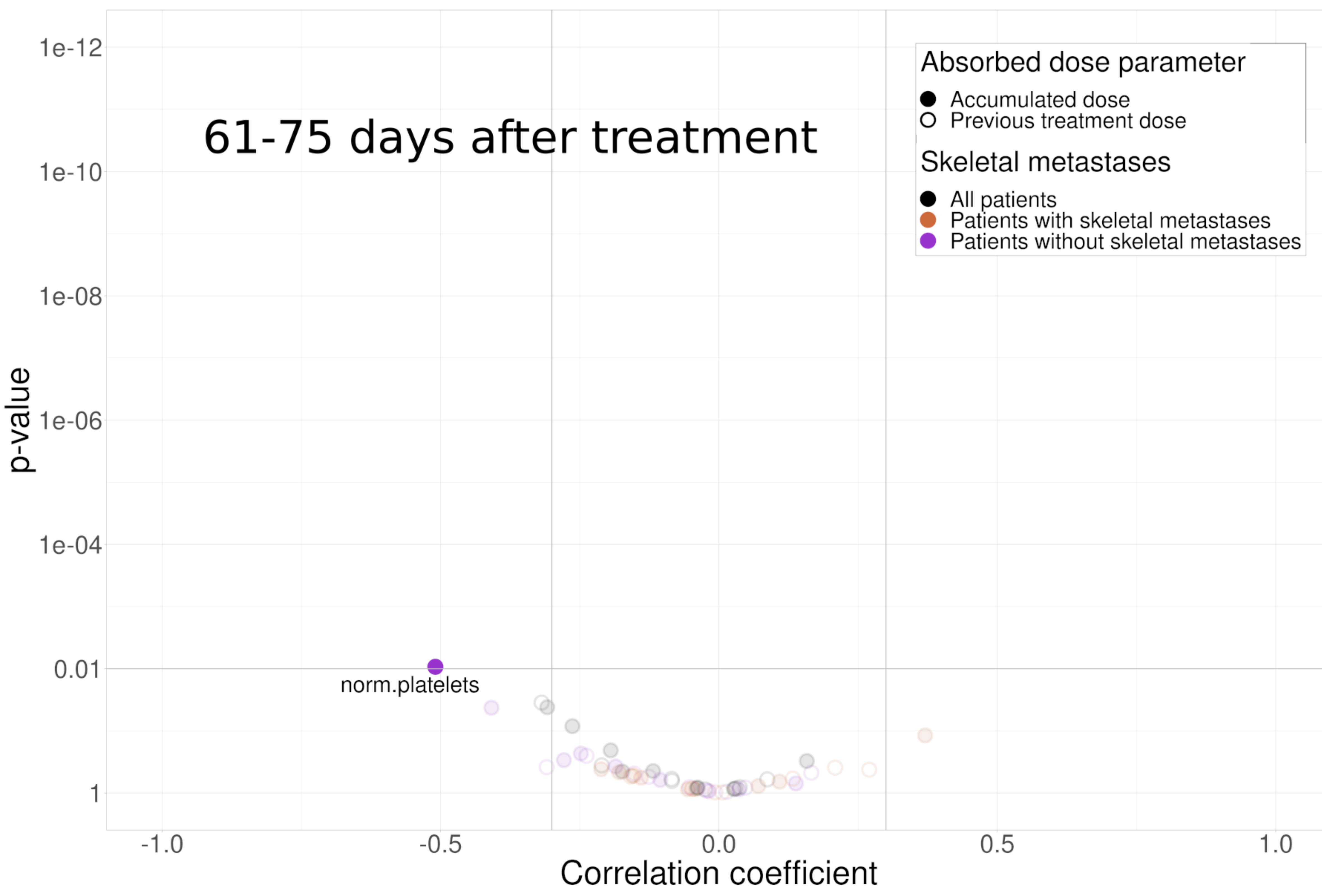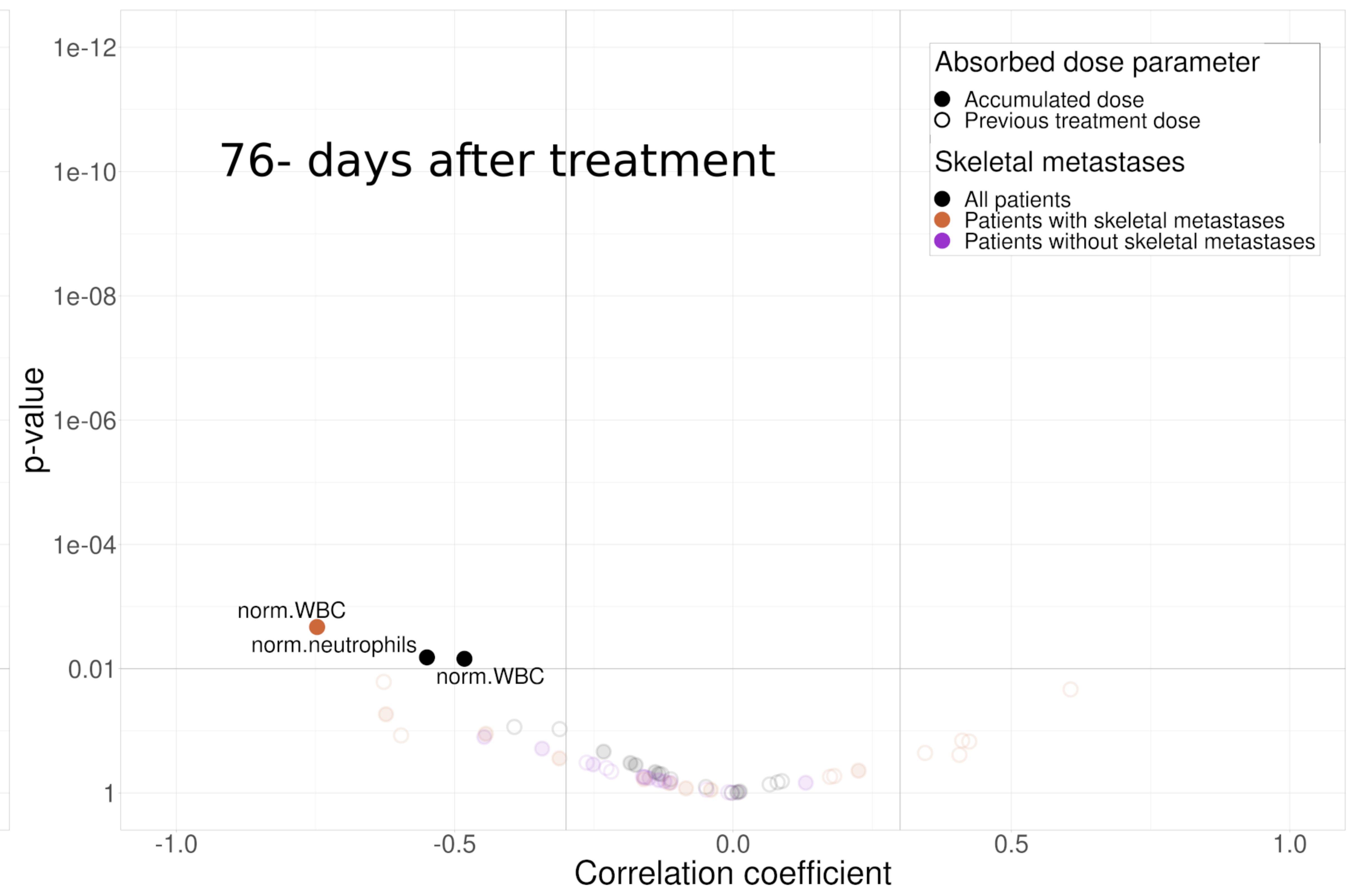

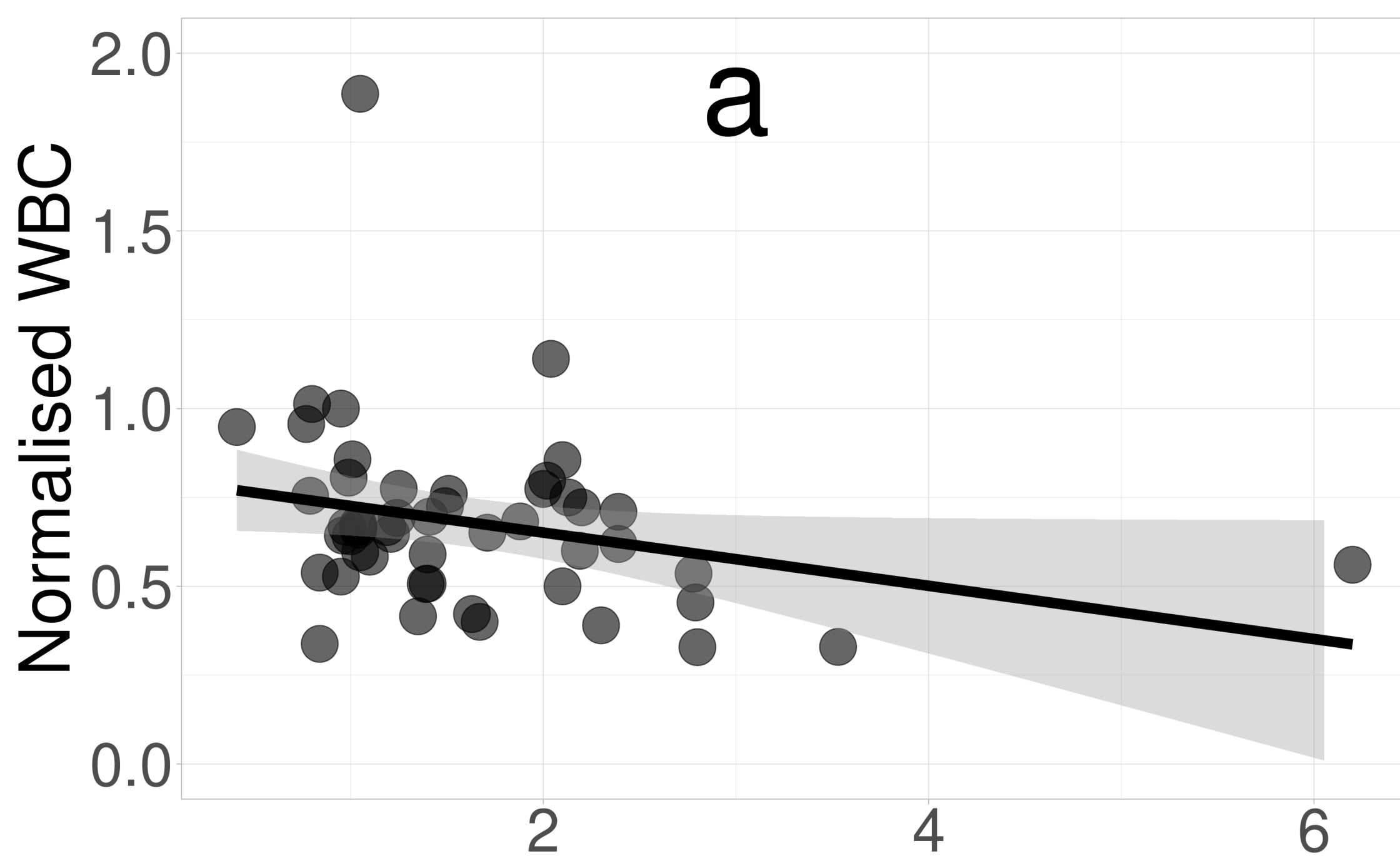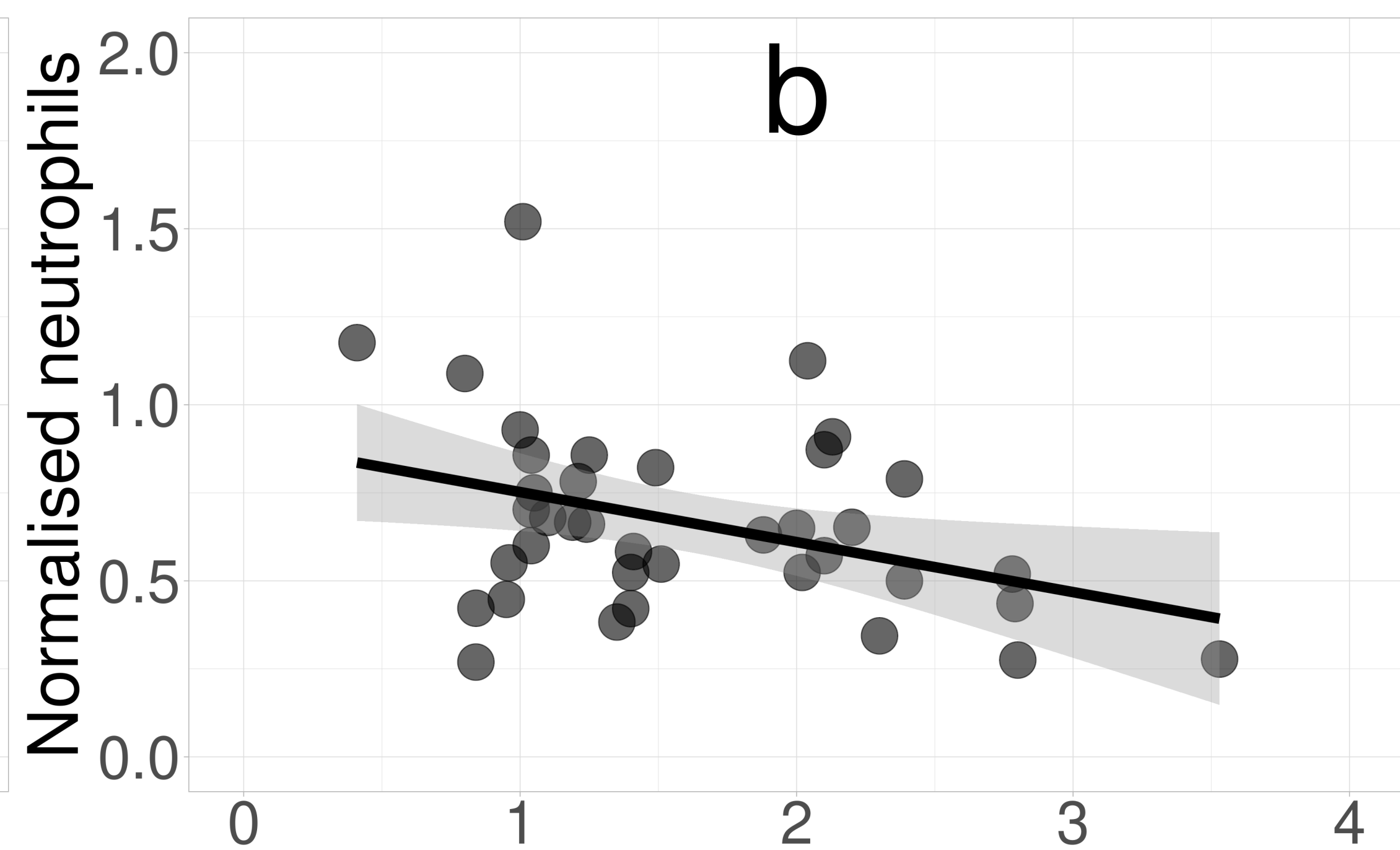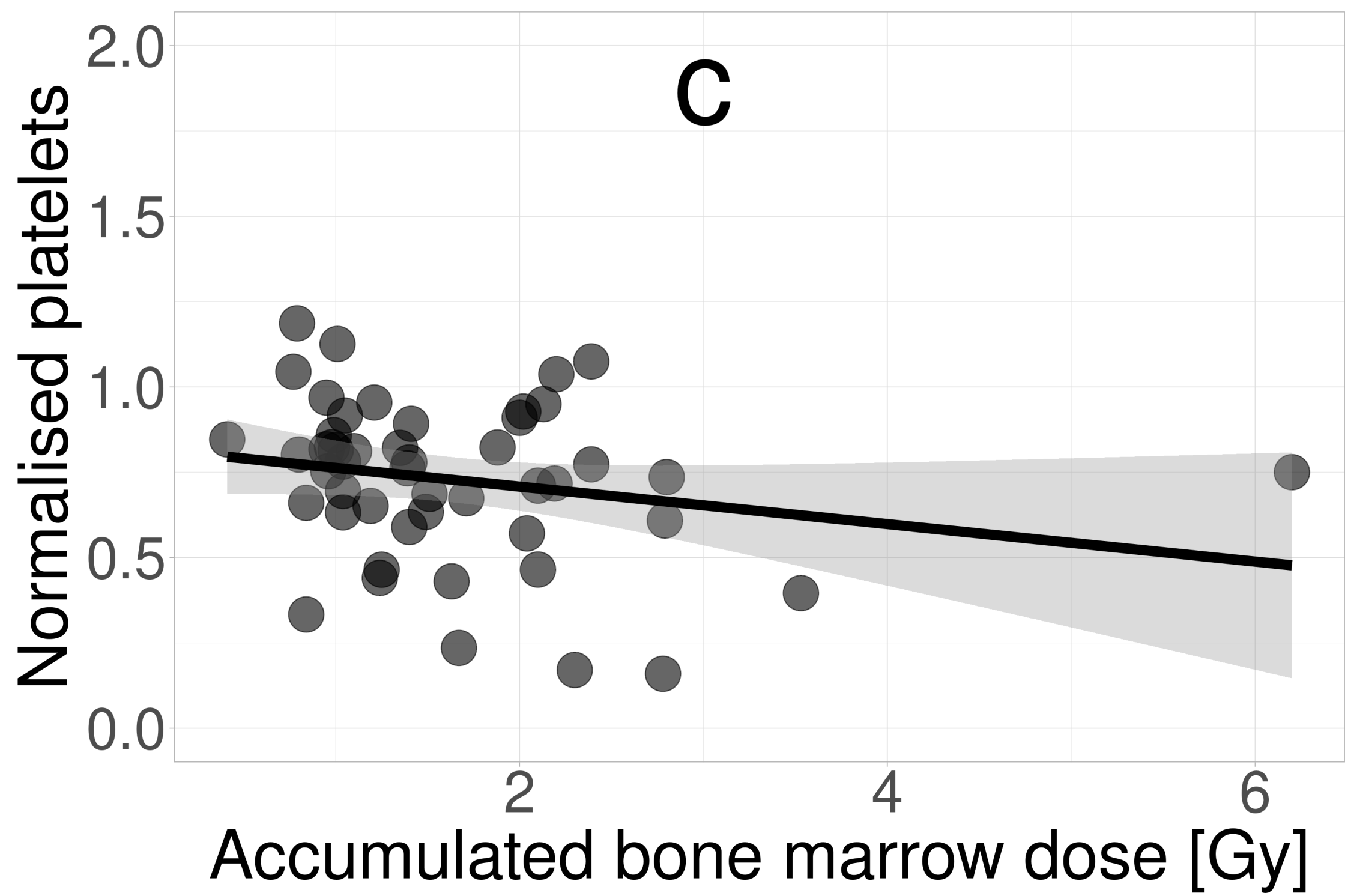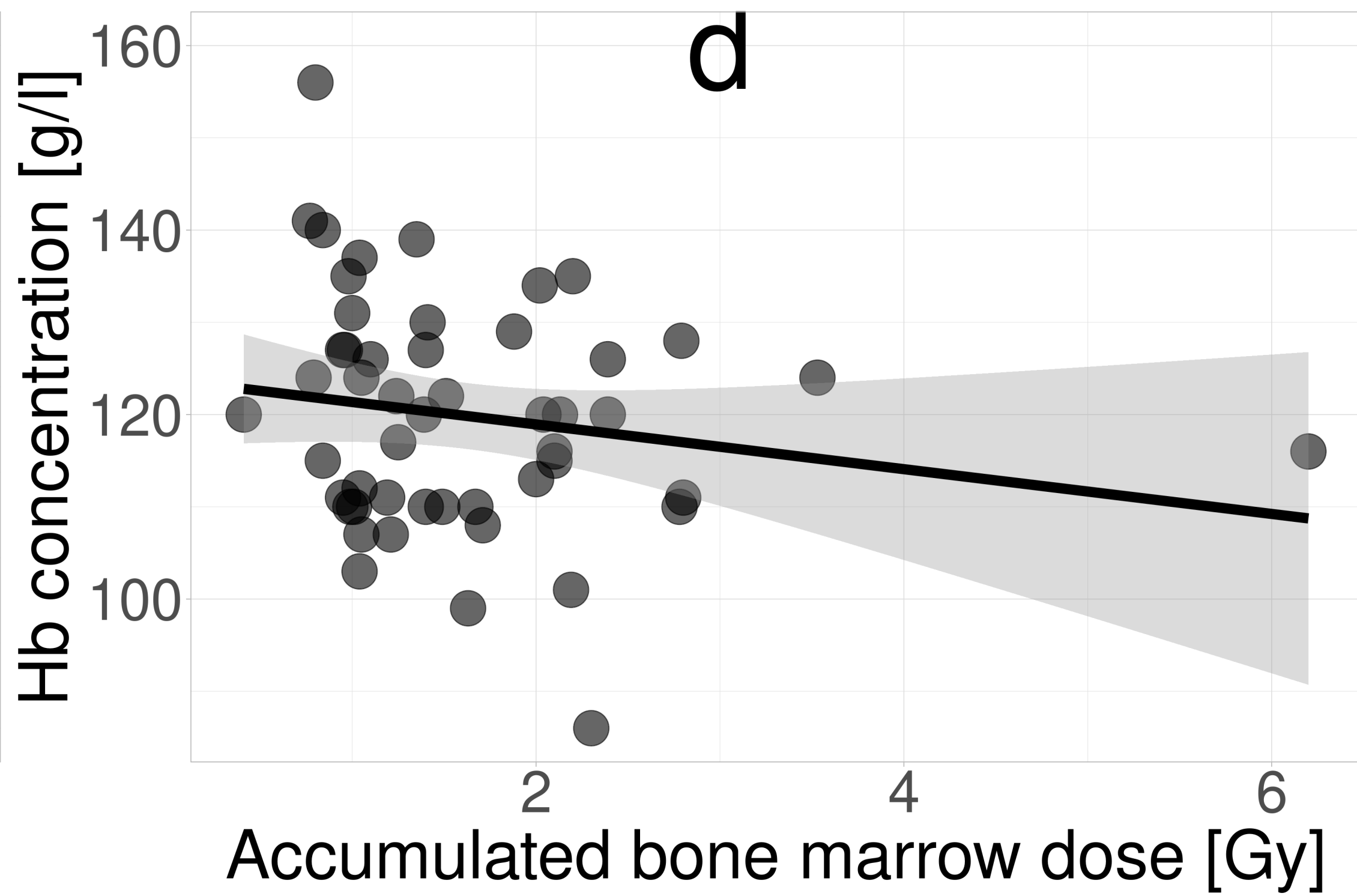

Supplement: Supplementary file 3 — Additional file 3. Volcano plot montage. [file 13550_2024_1077_MOESM3_ESM.pdf]
