# Supplementary material for: Risk of treatment-altering haematological toxicity and its dependence on bone marrow doses in peptide receptor radionuclide therapy
Source: EJNMMI Res. 2024 Feb 6;14:13. doi: 10.1186/s13550-024-01077-7 (PMC10847080; doi:10.1186/s13550-024-01077-7)
Supplement: Supplementary file 4 — Additional file 4. Correlation analysis with and without previously treated patients. [file 13550_2024_1077_MOESM4_ESM.pdf]

Supplementary material - Correlation analysis with and without previously treated patients

Correlations (> +/-0.3) found in analysis of all patients:

blood_parameter	correlation	CI_low	CI_high	pvalue	sampling_period
leukocyte_norm	-0.33	-0.45	-0.21	6.92E-07	1-15
leukocyte_norm	-0.36	-0.47	-0.23	1.91E-07	16-30
leukocyte_norm	-0.43	-0.53	-0.32	8.70E-12	31-45
leukocyte_norm	-0.48	-0.72	-0.15	6.93E-03	76-90
neutrophil_norm	-0.32	-0.46	-0.17	5.31E-05	16-30
neutrophil_norm	-0.41	-0.53	-0.28	1.41E-08	31-45
neutrophil_norm	-0.55	-0.78	-0.18	6.57E-03	76-90
thrombocyte_norm	-0.36	-0.47	-0.24	6.85E-08	1-15
thrombocyte_norm	-0.33	-0.45	-0.20	2.16E-06	16-30
thrombocyte_norm	-0.35	-0.46	-0.23	3.53E-08	31-45
thrombocyte_norm	-0.40	-0.54	-0.24	4.40E-06	46-60
Hb	-0.31	-0.42	-0.19	1.49E-06	31-45

Correlations (> +/-0.3) found in analysis of patients with no previous PRRT:

blood_parameter	correlation	CI_low	CI_high	pvalue	sampling_period
leukocyte_norm	-0.32	-0.44	-0.19	5.78E-06	1-15
leukocyte_norm	-0.35	-0.47	-0.21	1.58E-06	16-30
leukocyte_norm	-0.41	-0.52	-0.29	4.20E-10	31-45
leukocyte_norm	-0.51	-0.74	-0.16	7.07E-03	76-90
neutrophil_norm	-0.31	-0.45	-0.15	1.94E-04	16-30
neutrophil_norm	-0.39	-0.52	-0.25	3.88E-07	31-45
neutrophil_norm	-0.59	-0.81	-0.21	4.79E-03	76-90
thrombocyte_norm	-0.35	-0.47	-0.22	6.11E-07	1-15
thrombocyte_norm	-0.32	-0.44	-0.18	1.65E-05	16-30
thrombocyte_norm	-0.35	-0.46	-0.22	2.30E-07	31-45
thrombocyte_norm	-0.41	-0.55	-0.24	9.83E-06	46-60
thrombocyte	-0.33	-0.49	-0.15	4.60E-04	46-60
Hb	-0.30	-0.43	-0.16	4.09E-05	16-30
Hb	-0.32	-0.44	-0.20	1.64E-06	31-45
Hb	-0.30	-0.46	-0.12	1.46E-03	46-60
